# Risk Factors for Obstetric Anal Sphincter Injuries among Women Delivering at a Tertiary Hospital in Southwestern Uganda

**DOI:** 10.1155/2020/6035974

**Published:** 2020-05-14

**Authors:** Mahad Ali, Richard Migisha, Joseph Ngonzi, Joy Muhumuza, Ronald Mayanja, Jolly Joe Lapat, Wasswa Salongo, Musa Kayondo

**Affiliations:** ^1^Department of Obstetrics and Gynecology, Mbarara University of Science and Technology, Mbarara, P.O Box 1410, Uganda; ^2^Department of Physiology, Mbarara University of Science and Technology, Mbarara, P.O Box 1410, Uganda

## Abstract

**Background:**

Obstetric anal sphincter injuries (OASIS) arise from perineal trauma during vaginal delivery and are associated with poor maternal health outcomes. Most OASIS occur in unattended deliveries in resource-limited settings. However, even in facilities where deliveries are attended by skilled personnel, a number of women still get OASIS.

**Objectives:**

To determine the incidence and risk factors for obstetric anal sphincter injuries among women delivering at Mbarara Regional Referral Hospital (MRRH).

**Methods:**

We conducted an unmatched hospital-based case control study, with the ratio of cases to controls of 1 : 2 (80 cases and 160 controls). We defined a case as a mother who got a third- or fourth-degree perineal tear after vaginal delivery while the controls recruited were the next two mothers who delivered vaginally without a third- or fourth-degree perineal tear. A questionnaire and participants' medical records review were used to obtain sociodemographic and clinical data. We estimated the incidence of OASIS and performed univariable and multivariable logistic regression to identify the associated risk factors.

**Results:**

The cumulative incidence for OASIS during the study period was 6.6%. The risk factors for OASIS were 2^nd^ stage of labour ≥1 hour (aOR 6.07, 95%CI 1.86–19.82, *p*=0.003), having episiotomy performed during labour (aOR 2.57, 95%CI 1.07–6.17, *p*=0.035), perineum support during delivery (aOR 0.03, 95%CI 0.01–0.12, *p* < 0.001), and monthly income of >50,000 shillings (aOR 0.09, 95%CI 0.03–0.28, *p* < 0.001). *Conclusions and Recommendations*. The risk factors for obstetric anal sphincter injury were prolonged second stage of labour and performing episiotomies during deliveries while higher monthly income and perineum support during delivery were protective. We recommend routine support to the perineum during delivery. Care should be taken in mothers with episiotomies, as they can extend and cause OASIS.

## 1. Background

An obstetric anal sphincter injury (OASI) refers to third- and fourth-degree perineal tears. Third-degree tears involve a partial or complete disruption of the anal sphincter complex which includes the external anal sphincter and the internal anal sphincter. Fourth-degree tears involve disruption of the anal mucosa in addition to division of the anal sphincter complex [1]. Vaginal delivery is the major cause of anal dysfunction in women. Between 0.6% and 9.0% of women, who deliver vaginally, where mediolateral episiotomy is performed, sustain obstetric anal sphincter injuries (OASIS) [2].

A study in England found four-fold increase in the rate of reported third- or fourth-degree perineal tears, with the rate rising from 1.8% in 2000 to 5.9% in 2011 [3]. Obstetric anal sphincter injuries cause significant morbidity including anal incontinence, rectovaginal fistula, and pain [1]. Anal incontinence is the most distressing and disabling of the OASIS complications. Anal incontinence includes a variety of symptoms including passive soiling, flatus incontinence, and incontinence of liquid or solid stool [4]. Hence, OASIS negatively impacts on women's health by impairing their quality of life in both the short and long term. One of the most distressing immediate complications of perineal injury is perineal pain, which can result into urinary retention and defecation problems in the immediate postpartum period. In the long term, women with perineal pain may have dyspareunia and altered sexual function. Additionally, complications of severe perineal tears include abscess formation, wound breakdown, and rectovaginal fistulae [1]. Furthermore, outcomes following repair of OASIS are still very poor. For instance, rates of anal incontinence following the primary repair of OASIS ranging from 15% to 60%, with an average of 39%, have been reported [5]. Therefore, understanding the modifiable risk factors for OASIS is key in identifying interventions to prevent the occurrence of the perineal trauma and associated maternal complications.

The true magnitude of OASIS is believed to be under-reported because of fear of litigation. This is particularly true in Sub-Saharan Africa, where the reported incidence in the region ranges between 1% and 11% [6]. Maternal age above 25 years, forceps and ventouse delivery, especially without episiotomy, Asian ethnicity, a more affluent socioeconomic status, higher birth weight, and shoulder dystocia have been reported to be associated with increased risk for OASIS [2]. Use of epidural analgesia was found to be protective, while use of vacuum extraction without a prior episiotomy, fetal weight greater than 4 kg, and fetal occipital posterior positions were associated with increased risk for OASIS [7].

There are limited data on the burden and risk factors of OASIS in southwestern Uganda. Most OASIS are thought to occur in unattended deliveries in low-income countries. However, there is evidence to suggest that even in deliveries that are attended by skilled health personnel, such as is the case in referral and teaching hospitals, OASIS still occur. This study therefore aimed to determine the incidence and risk factors of OASIS among women delivering at Mbarara Regional Referral Hospital (MRRH) in southwestern Uganda.

## 2. Methods

### 2.1. Study Population and Setting

This study was conducted on the maternity ward of Mbarara Regional Referral Hospital (MRRH) from February to May 2019. MRRH is a tertiary public health hospital providing services to ten districts in southwestern Uganda (Mbarara, Bushenyi, Ibanda, Isingiro, Kiruhura, Buhweju, Mitooma, Rubirizi, Sheema, and Ntungamo), with an estimated catchment population of about 5 million people. The hospital also serves as a teaching hospital for postgraduates and undergraduate medical students of the Mbarara University of Science and Technology and is an internship training site for healthcare professionals in the country. In addition, MRRH is a training site for nursing, midwifery, and clinical school students of other health training institutions such as Mayanja Memorial Hospital and Bishop Stuart University. The maternity ward records about 12,000 deliveries annually, with an average of 20 to 30 deliveries per day.

We conducted a hospital based unmatched case control study from 27^th^ February 2019 to 22^nd^ May 2019. We defined a case as a mother who got a third- or fourth-degree perineal tear after vaginal delivery. We included all women who delivered vaginally at MRRH and excluded those with preexisting unrepaired third- and fourth-degree perineal tears. Consecutive sampling of all mothers who met the inclusion criteria was performed to recruit the cases. The controls recruited were the next two mothers who delivered after a case. If a case was followed by a case, then the next four controls that followed were recruited. The diagnosis of OASIS was confirmed by an attending consultant obstetrician on duty to ensure quality control. We defined third-degree perineal tears as tears involving the anal sphincter complex, while fourth-degree perineal tears included tears involving both the anal sphincter complex and the anal epithelium [8].

### 2.2. Data Collection and Study Variables

A semistructured questionnaire was administered to capture clinical and sociodemographic data. We also reviewed the medical records of the participants to capture obstetric details. The outcome variable was the occurrence of third- and fourth-degree perineal tear (OASIS) among women delivering at Mbarara Regional Referral Hospital. The exposure variables included maternal sociodemographic characteristics, obstetric factors, and intrapartum factors. The maternal sociodemographic variables included age, education level, level of income, occupation, marital status, tribe, religion, and body mass index. The maternal obstetric variables included gestation age at delivery, gravidity, attendance of antenatal care, weight of babies delivered, and induction of labour. Intrapartum variables included induction of labour, augmentation of labour, duration of labour, stage of labour at admission, duration of second-stage labour, cadre of birth attendants, instrumental delivery, episiotomy use, presentation of the baby, position of the fetal head, head circumference of the baby, and the HIV status of the mother.

### 2.3. Sample Size and Statistical Analysis

We calculated the sample size using OpenEpi, based on the formula described by Kelsey et al [9] on the basis of the following assumptions: two-sided confidence level of 95%; power of 80%; ratio of controls to cases of two; 37.6% of the controls exposed; 52.7% of cases exposed and least extreme odds ratio to be detected of 2.17. This gave us a total sample size of 240 participants (80 cases and 160 controls). Our exposure of interest was parity. The information on the proportion of cases and controls exposed was sourced from the study conducted in South Africa, in which 52.7% of the women who had third- and fourth-degree perineal tear were prime gravidas and among 37.6% of the controls were prime gravidas [10].

Data were manually entered in EpiData 3.1 software (EpiData, Odense, Denmark) and then exported to STATA version 13 (StataCorp, College Station, Texas, USA) for analysis. Data on demographic, social, obstetric, clinical, and intrapartum characteristics were compared by case status using chi square (*χ*2) test and Fischer's exact tests. Associations were quantified with univariable and multivariable logistic regression. Differences between nonparametric variables (expressed as median, range) were compared using the Wilcoxon rank-sum test. Variables associated with *p* value ≤0.2 in the univariable analysis were entered into the multivariable logistic regression model through a backward stepwise elimination method to obtain the final predictive model of independent risk factors for OASIS with *p* < 0.05.

The incidence of OASIS among vaginal deliveries was obtained by dividing the number of cases of OASIS by the total number of vaginal deliveries observed during the study period and expressed as a percentage.

### 2.4. Ethical Considerations

The study was approved by the Faculty of Medicine Research Committee of the Mbarara University of Science and Technology. We also got approval from the Mbarara University of Science and Technology Research and Ethics Committee (MUST-REC). Our study was assigned no.06/01-19 by the MUST-REC. All study participants provided written informed consent before recruitment and participation. Participants who could not write gave consent with a thumbprint. We respected the Declaration of Helsinki guidelines and CIOMS-2002 (Council for International Organizations of Medical Sciences) regarding research with humans, avoiding any type of physical or moral damage.

## 3. Results

### 3.1. Baseline Sociodemographic Characteristics of the Study Participants

The sociodemographic characteristics are presented in Table 1. We recruited a total of 240 (80 cases and 160 controls) participants, with a mean age of 24.9 years (SD ± 5.4). Majority of the participants had ever attained formal education (96.3%), were married (91.7%), were Banyakole by tribe (75.4%), and earned less than 50,000 Ugandan shillings monthly (70.0%). The proportion of participants above 25 years was significantly more in cases compared to controls, *p*=0.003. Similarly, the proportion of participants who earned less than 50,000 shillings monthly was significantly more in cases than in controls, *p*=0.001.

The baseline obstetric and clinical factors are presented in Table 2. Majority of the participants were multigravida (60.7%), had ever attended antenatal care (97.9%), delivered during day time (64.4%), had their perineum supported during delivery (83.8%), and had spontaneous vertex mode of delivery (97.4%). The median duration of second stage of labour was 25 (IQR 10, 40) minutes. Induction of labour was performed in 16 participants (7.0%) overall. Episiotomy was performed in 73/223 (32.7%) participants. A total of 34/237 (14.4%) participants were HIV positive. As shown in Table 2, the proportion of prime gravidas with OASIS (62.5%) was significantly more compared to controls (27.7%), *p* < 0.001. Also, the proportion of participants with duration of second stage of labour ≥1 hour was statistically higher among cases (23.8%) compared to controls (6.0%), *p* < 0.001.

### 3.2. Risk Factors for OASIS in Univariable Analysis

As can be seen in Table 3, the categories of participants that were more likely to develop OASIS were prime gravidas, those whose duration of labour lasted ≥1 hour and those in whom episiotomies were performed. On the contrary, mothers aged >25 years, those whose perineum was supported during delivery and those who earned more than 50,000 Ugandan shillings monthly were protective against developing OASIS.

### 3.3. Risk Factors for OASIS in Adjusted Regression Analysis

In multivariable logistic regression analysis, the risk factors that were significantly associated with developing OASIS were second stage of labour ≥1 hour (aOR 6.07, 95%CI 1.86–19.82, *p*=0.003) and having episiotomy performed during delivery (aOR 2.57, 95%CI 1.07–6.17, *p*=0.035). Perineum support during delivery (aOR 0.03, 95%CI 0.01–0.12, *p* < 0.001) and earning more than 50,000 Ugandan shillings (aOR 0.09, 95%CI 0.03–0.28, *p* < 0.001) were protective against OASIS.

### 3.4. Incidence of OASIS

**Figure 1:**
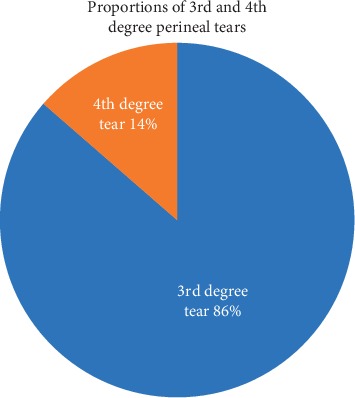
The proportions of third- and fourth-degree perineal tears among participants with OASIS during the study period.

The total number of vaginal deliveries during the study period from 27^th^ February to 22^nd^ May 2019 was 1,220 of which 80 had OASIS. This gave us the incidence rate of 6.6% (95%CI 5.2–8.1%) among women delivering at the maternity ward of MRRH. Out of the 80 cases with OASIS, 70 (86%) had third-degree perineal tears as shown in Figure 1.

## 4. Discussion

The incidence of OASIS of 6.6% found in this study compares with the overall risk rate of 6.3% documented in a meta-analysis [11]. However, it was higher than the rates reported elsewhere in high-income countries. For instance, incidence rates of less than 5% have more recently been reported in Hong Kong, Ireland, USA, United Kingdom, Norway, and Sweden [12–16]. We postulate that the variation in the incidence could be arising from the different study designs employed. For example, the study in Ireland was conducted in two tertiary referral hospitals for a period of 8 years, while the one in the USA was a population-based study. Our study considered one referral hospital in period of 3 months.

In an adjusted regression model, the risk factors for OASIS were having had an episiotomy performed and prolonged 2^nd^ stage of labour. Higher monthly income (>50,000 shillings) and having perineum supported during delivery were protective against OASIS. The association between prolonged duration of 2^nd^ stage of labour and increased risk for OASIS found in the current study is consistent with prior findings [13, 17, 18]. This finding has important clinical and public health implications, as current obstetric practice now encourages longer duration of second stage of labour in an effort to decrease the escalating number of primary caesarian section deliveries [19]. In the light of our findings, therefore, such decisions should be weighed against the risk for OASIS. On the contrary, a case control study in a tertiary hospital in South Africa found no association between duration of second stage of labour and third- or fourth-degree perineal tear [10].

We found that performing episiotomy was a risk factor for OASIS. This is in agreement with previous studies that have reported women with episiotomies to be at an increased risk for OASIS [20, 21]. This is thought to be due to the fact that episiotomy incisions may extend, particularly for midline episiotomies [22]. In our study, we did not adjust for the episiotomy technique employed, since the details of the type of episiotomy performed were not captured. This may have resulted into residual confounding. It is possible that other factors, such as the professional cadre that performed the episiotomy, the angle of the episiotomy, and the indications for performing the episiotomies (e.g., difficult deliveries), could have conferred the risk of OASIS, rather than performing episiotomy itself. Nonetheless, our findings do not support routine indiscriminate use of episiotomies during vaginal deliveries.

Manual support of perineum during labour was protective against OASIS in the current study. This is consistent with the study which evaluated the risk of OASIS after an intervention of manual support of the perineum during the expulsive phase of second-stage labour in which the risk of sustaining OASIS decreased by 59% after the intervention [23]. In another study in Norway, manual perineal support during labour was also found to reduce the overall risk of OASIS, with greatest protection seen in prime parous women [24]. Perineal support is believed to slow down the speed of the presenting part through the vaginal canal, thus spreading the force evenly on the perineum [24]. In addition, smooth, controlled perineal support of the head delivery and its association with less perineal trauma has been well documented [25, 26]. Therefore, our findings further highlight the importance of encouraging delivery positions that enable clear visualization of the perineum so that the health workers are in a better position to support the perineum. On the contrary, one meta-analysis of randomized controlled trials on manual perineal support during delivery reported no significant decrease in risk of OASIS [27].

Our study found prime gravidas at an increased risk for OASIS. Indeed, parity is a well-known risk factor for third- and fourth-degree perineal tears [19, 28–30]. The most biologically plausible mechanism is the limited elasticity of the perineum among nulliparous and prime gravida women compared to multigravidas [31]. This finding calls for high index of suspicion for OASIS among prime gravidas so as to prevent their occurrence and/or facilitate their early recognition and prompt repair. There is evidence to suggest that a good number of OASIS go undetected by midwives and junior doctors [32].

Lastly, we found that women who had higher monthly income (>50,000 Ugandan shillings) were protected against OASIS. This is a new finding. We suspect this is because women with higher income in our setting in Uganda are in better position to seek appropriate obstetric care timely. For instance, they are less likely to get prolonged labour from delays to reach health facilities that could arise from lack of transport.

Our study has some limitations. Firstly, recall bias may arise from the case control design of our study, as participants were asked for information about some exposures that had taken place a long period (more than twelve months) preceding the interview date. This was minimized by giving participants ample time to recall. Secondly, the fact that our study was conducted in only one hospital may limit the generalizability of our findings to the populations beyond the tertiary hospital setting. Lastly, we used medical records to obtain some data on obstetric factors and this led to some missing data on some variables, for some participants who were discharged earlier than anticipated. However, the missing data are very unlikely to bias our findings, since the data were missing completely at random.

## 5. Conclusions

The incidence rate of 6.6% is higher than what most studies have documented. Prolonged second stage of labour and performing episiotomies were associated with increased risk for OASIS. Higher income and manual support of the perineum were protective against OASIS. We recommend routine perineal support and restrictive use of episiotomies during delivery. Additional care should be taken in women with episiotomies as they can extend and cause OASIS.

## Figures and Tables

**Table 1:** Baseline sociodemographic characteristics of study participants by case status.

Characteristic		Overall (*N* = 240)	Cases (*N* = 80)	Controls (*N* = 160)	*p* value
		n/N (%)	n/N (%)	n/N (%)	
Age, years, mean (SD)		24.9 (±5.4)	23.3 (±3.6)	25.7 (±5.9)	0.001
Age category (years)					
≤ 25		148 (61.7)	60 (75.0)	85 (55.0)	
>25		92 (38.3)	20 (25.0)	72 (45.0)	0.003
Occupation					0.649
Housewife		93 (38.8)	32 (40.0)	61 (38.13)	
Business woman		69 (28.75)	20 (25.0)	49 (30.63)	
Other occupation		78 (32.5)	28 (35.0)	50 (31.25)	
Education category					0.138
None		9 (3.8)	1 (1.25)	8 (5.00)	
Primary		115 (47.9)	35 (40.7)	80 (52.0)	
Secondary		93 (38.8)	38 (47.5)	55 (34.4)	
Tertiary		23 (9.6)	8 (10.0)	15 (9.4)	
Marital status					0.248
Single		20 (8.3)	9 (11.5)	11 (6.88)	
Married		220 (91.7)	71 (88.75)	149 (93.13)	
Religion					0.267
Catholic		90 (37.5)	34 (42.5)	56 (35.0)	
Protestant		104 (43.3)	35 (43.13)	69 (43.13)	
Others		35 (21.88)	11 (13.75)	46 (19.17)	
Tribe					0.244
Munyakole		181 (75.4)	64 (80.0)	117 (73.13)	
Others		59 (24.6)	16 (20.0)	43 (26.8)	
Income in Ugx (*N* = 227)					0.001
50.000 or less		159 (70.0)	76 (95.0)	83 (56.46)	
More than 50.000		68 (30.0)	4 (5.0)	64 (43.54)	
Referred, yes		78 (32.6)	29 (36.76)	49 (30.63)	0.345
Body mass index, kg/m^2^, mean (SD)		25.2 (±3.7)	25.0 (±3.2)	25.3 (±4.0)	0.567
Body mass index, kg/m^2^ (*N* = 235)					0.593
Below 25		126 (53.6)	44 (55.0)	82 (52.9)	
25–29.9		86 (36.6)	30 (37.5)	56 (36.13)	
30 and above	23 (9.8)	6 (7.50)	13 (10.97)	

SD = standard deviation; *k* = 1,000 shillings; Ugx = Ugandan shillings.

**Table 2:** Baseline obstetric and clinical factors of study participants by case status.

Characteristic	Overall (*N* = 240)	Cases (*N* = 80)	Controls (*N* = 160)	*p* value
	n/N (%)	n/N (%)	n/N (%)	
Gravidity (*N* = 239)				
Primigravida	94 (39.3)	50 (62.5)	44 (27.7)	<0.001
Multigravida	145 (60.7)	30 (37.5)	115 (72.3)	
ANC attendance, yes (*N* = 239)	234 (97.9)	77 (97.5)	157 (98.1)	0.739
Gestational age at delivery (*N* = 239)				0.001
<37 wks.	6 (2.5)	1 (1.2)	5 (3.1)	
37–42 wks.	192 (80.3)	75 (93.8)	117 (73.6)	
>42 weeks	41 (17.15)	4 (5.0)	37 (23.3)	
Time of delivery (*N* = 233)				0.967
Day time	150 (64.4)	51 (64.6)	99 (64.3)	
Night time	83 (35.6)	28 (35.4)	55 (35.7)	
Duration of 2nd stage of labour, minutes, median (IQR)	25 (10, 40)	30 (20, 53)	20 (10, 30)	0.001
Duration of 2nd stage of labour (*N* = 234)				<0.001
<1 hour	205 (87.6)	64 (76.2)	141 (94.0)	
≥1 hour	29 (12.4)	20 (23.8)	9 (6.0)	
Labour induction, yes (*N* = 229)	16 (7.0)	5 (6.7)	11 (7.1)	0.894
Labour augmentation, yes (*N* = 207)	19 (9.2)	6 (9.1)	13 (9.2)	0.976
Episiotomy performed, yes (*N* = 223)	73 (32.7)	33 (45.8)	40 (26.5)	0.004
Perineum supported, yes (*N* = 228)	191 (83.8)	42 (58.3)	149 (95.5)	0.001
Presentation (*N* = 239)				1.000
Cephalic	238 (99.6)	80 (100.0)	158 (99.4)	
Breech	1 (0.4)	0 (0.0)	1 (0.6)	
Position (*N* = 237)				1.000
Occipital anterior	235 (99.2)	79 (100.0)	156 (98.7)	
Occipital posterior	1 (0.4)	0 (0.0)	1 (0.6)	
Sacrum anterior	1 (0.4)	0 (0.0)	1 (0.6)	
Mode of delivery (*N* = 233)				0.829
Spontaneous vertex	227 (97.4)	77 (96.5)	150 (97.4)	
Vacuum extraction	4 (1.7)	2 (2.5)	2 (1.3)	
Spontaneous breech	1 (0.4)	0 (0.0)	1 (0.7)	
Assisted breech	1 (0.4)	0 (0.0)	1 (0.7)	
Head circumference, cm, mean (SD)	36.6 (±1.9)	36.6 (±1.9)	36.6 (±1.9)	0.912
HIV status, positive (*N* = 237)	34 (14.35)	10 (12.7)	24 (15.2)	0.836

**Table 3:** Risk factors for anal sphincter injuries (OASIS) in multivariable and univariable logistic regression analyses.

Characteristic	% of cases	Univariable analysis	*p* value	Multivariable analysis	*p* value
	n/N (%)	OR (95%CI)		Adjusted OR (95%CI)	
Age category (years)					
≤ 25	60/148 (40.5)	Ref	0.003	Ref	0.963
>25	20/92 (21.7)	0.41 (0.22–0.74)		0.98 (0.40–2.39)	
Income in Ugx					
50 k or less	76/159 (47.8)	Ref	0.001	Ref	<0.001
More than 50 k	4/68 (5.9)	0.07 (0.02–0.20)		0.09 (0.03–0.28)	
Gravidity					
Multigravida	30/145 (20.7)	Ref	0.001	Ref	0.068
Prime gravida	50/94 (53.2)	4.35 (2.46–7.71)		2.40 (0.94–6.14)	
Duration of 2nd stage of labour					
<1 hour	64/205 (31.2)	Ref	<0.001	Ref	0.003
≥1 hour	20/29 (69.0)	4.90 (2.11–11.34)		6.07 (1.86–19.82)	
Performed episiotomy					
No	26/150 (26.0)	Ref	0.004	Ref	0.035
Yes	33/73 (45.2)	2.35 (1.30–4.23)		2.57 (1.07–6.17)	
Perineum supported					
No	30/37 (81.1)	Ref	0.001	Ref	<0.001
Yes	41/191 (22.0)	0.07 (0.03–0.16)		0.03 (0.01–0.12)	

*K* = 1,000 shillings; Ugx = Ugandan shillings; Ref = reference category.

## Data Availability

The datasets generated and analyzed during the study are available from the corresponding author upon request.
